# Improving Glycemic Control With a Standardized Text-Message and Phone-Based Intervention: A Community Implementation

**DOI:** 10.2196/diabetes.7910

**Published:** 2017-07-25

**Authors:** Robert Mattson Peters, Matt Lui, Kunjan Patel, Lewis Tian, Kavon Javaherian, Eric Sink, Ran Xu, Zhuchen Xu, Wint Aung, Li Zhou, Justin Huynh, Gregory Polites, Melvin Blanchard, Avik Som, Will Ross, Carlos Bernal-Mizrachi

**Affiliations:** 1 Washington University in St. Louis School of Medicine St. Louis, MO United States; 2 Saint Louis University School of Medicine St. Louis, MO United States; 3 John Cochrane Division VA Medical Center St. Louis, MO United States; 4 Division of Internal Medicine Mercy Clinic St. Louis, MO United States; 5 Epharmix, Inc St. Louis, MO United States; 6 Division of Endocrinology St. Louis VA Medical Service St. Louis, MO United States; 7 Division of Endocrinology Metabolism and Lipid Research Washington University in St. Louis School of Medicine St. Louis, MO United States

**Keywords:** diabetes mellitus, glycemic control, telemedicine, electronic health (eHealth), mobile health (mHealth), telehealth, SMS, diabetes management

## Abstract

**Background:**

Type II diabetes mellitus (T2DM) presents a major disease burden in the United States. Outpatient glycemic control among patients with T2DM remains difficult. Telemedicine shows great potential as an adjunct therapy to aid in glycemic control in real-world settings.

**Objective:**

We aimed to explore the effectiveness of EpxDiabetes, a novel digital health intervention, in improving hemoglobin A1c (HbA1c) and fasting blood glucose (FBG) among patients with uncontrolled diabetes.

**Methods:**

We recruited 396 patients from a community clinic in St. Louis, Missouri, from a database of patients diagnosed with T2DM and with a most recent HbA1c >7% as part of a quality improvement project. An automated call or text-messaging system was used to monitor patient-reported FBG. If determined to be elevated, care managers were notified by email, text, or electronic medical record alert. Participants self-reported their FBG data by replying to EpxDiabetes automated phone calls or text messages. Data were subsequently analyzed, triaged, and shared with providers to enable appropriate follow-up and care plan adjustments. Absolute HbA1c reduction, patient engagement, and absolute patient-reported FBG reduction were examined at approximately 6 months post implementation.

**Results:**

EpxDiabetes had an average 95.6% patient response rate to messages at least once per month and an average 71.1% response rate to messages at least once per week. Subsequent HbA1c drop with EpxDiabetes use over 4 months was -1.15% (95% CI -1.58 to -0.71) for patients with HbA1c >8% at baseline compared to the change in HbA1c over 4 months prior to the implementation of EpxDiabetes of only -0.005 points (95% CI -0.28 to 0.27), *P*=.0018.

**Conclusions:**

EpxDiabetes may help reduce HbA1c in patients with high HbA1c baselines (>8%). The intervention demonstrates high patient engagement sustainable for at least 6 months.

## Introduction

Glycemic control among patients with type 2 diabetes mellitus (T2DM) remains a pressing problem ,. Poor glycemic control may be a factor of both poor access to care, poor health literacy, and poor return to follow-up and communication to providers of current glucose values. Current strategies to achieve glycated hemoglobin (HbA1c) control prove inadequate for a large proportion of patients ,. The standard of care for outpatient glycemic monitoring is a paper blood glucose log, but only 20%-52% of patients remain engaged with this tracking method, suggesting that there is a need to develop methodologies to improve patient follow-up and prevent complications [1-3].

Telemedicine interventions may facilitate remote glycemic monitoring, promote patient-provider communication, and reduce time to glycemic control. Most systems that monitor patients’ fasting blood glucose (FBG) use mobile phone apps, Internet-linked glucose monitors, or other specialized equipment [1-5]. Significant obstacles hinder the widespread dissemination and adoption of these technologies, especially among the elderly, those with low socioeconomic status, and those with low technological literacy [6,7]. Drop-out rates of up to 50% are reported in some studies using these modalities [8-10]. In the United States, most current telemedicine interventions involve one-way communication from provider to patient, or bidirectional systems that do not align with physician workflow or are not scalable ,1. Community implementation of these services can often be difficult due to logistics in implementing device-based solutions and feasibly analyzing patient-reported data within busy primary care practices. Therefore, there is a need for an intervention that is not only successful at engaging patients with low income and high HbA1c but also facilitates provider follow-up by providing triaged FBG data to close the patient-provider loop with improved patient follow-up.

To this aim, we developed , a novel bidirectional communication system designed to both collect patient FBG data and facilitate provider feedback to patients using smartly triaged FBG data in an overall low overhead implementation. The system utilizes ubiquitous text messaging technology or phone calls to collect FBG data as self-reported by patients and to identify dysglycemic trends and events [1 1. Providers are subsequently able to access the triaged data to provide necessary feedback to patients. Using this bidirectional feedback loop of communication, EpxDiabetes ultimately aims to accelerate HbA1c and FBG reduction by allowing earlier detection and provider intervention during dysglycemic events and trends. EpxDiabetes creates a closed feedback loop between patient and providers to achieve successive, rapid improvements in glycemic control.

To investigate the feasibility and effectiveness of the EpxDiabetes intervention in reducing HbA1c among patients with poor glycemic control in a real-world clinical setting, we conducted a proof-of-concept community implementation of the system.

## Methods

### Intervention Design

The EpxDiabetes intervention is part of a broader telemedicine platform, Epharmix, which has been developed and applied for the management of other diseases. The EpxDiabetes intervention modifies the existing platform to incorporate questions exclusive and specific to diabetic care. Similar to other Epharmix interventions, EpxDiabetes creates a two-way communication modality to enable providers to more quickly titrate and address problems [11]. In the focus with diabetes, the platform was able to generate a custom tool for glycemic control demonstrating the ability to actually modulate biometrics. Because populations with limited health literacy require interventions with simple, easy-to-read instructions , all EpxDiabetes messages read at a 4th grade level as determined by the Flesch-Kincaid Grade Level formula and calculated on the Readable.io website. Text messages and phone calls were provided free of charge (excluding standard messaging rates) to patients on any network to further promote accessibility among low socioeconomic populations.

EpxDiabetes consists of either automated phone calls or short-message service (SMS) messages sent to the patient’s preferred phone number, requesting patients to self-report their FBG values. The EpxDiabetes software algorithm remotely and automatically monitors patient-reported FBG values for hypoglycemic or hyperglycemic trends (FBG>160) or acute events (FBG<70 or >400). The frequency of messages for each patient varies based on their self-reported FBG to minimize message fatigue. Patients reporting dysglycemia receive messages more frequently than patients with euglycemia. In accordance with the definition in the 2013 report from the Workgroup on Hypoglycemia assembled by the American Diabetes Association and the Endocrine Society, hypoglycemia is defined in the EpxDiabetes system as a blood glucose value ˂70 mg/dL [12].

If the patient reports an FBG value beyond set thresholds, the designated provider receives an actionable “alert” notification via phone, SMS, or email requesting them to follow up with the patient. Any patient reporting hyperglycemia or hypoglycemia is provided the option to voluntarily contact their provider or to call 911 in the case of an emergency to minimize provider liability. When calling in, a patient hears the standard message, “If this is an emergency, please hang up and call 911” before being connected to their provider. The bidirectional patient-reporting and provider feedback loop represents a novel framework designed to achieve successive, rapid improvements in glycemic control.

**Figure 1:**
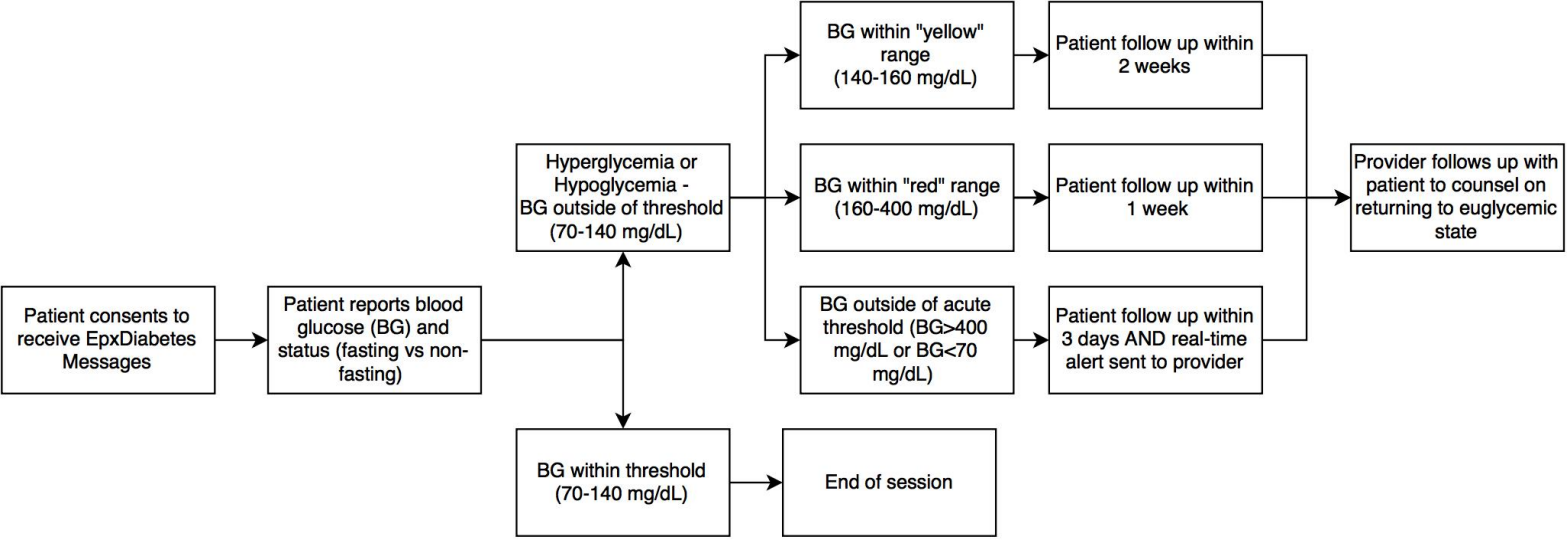
EpxDiabetes intervention flow diagram.

In addition to alert notifications, providers also receive a triaged bimonthly report prioritized by each patient’s average FBG values for longitudinal monitoring. The goal of the triaging system is to allow efficient review of overall population and individual patients and to facilitate selective attention to patients with dysglycemia (Figure 1).

### Patient Recruitment

The study was implemented as a prospective single-arm quality improvement project at community clinics across the St. Louis, Missouri, region. Patients consented to use the service under standard of care guidelines. Data were aggregated and de-identified per best practices for analysis under the permission of the community health center. A list of eligible participants was obtained by querying the clinic’s electronic medical record (EMR) using International Statistical Classification of Diseases and Related Health Problems (ICD)-9 and ICD-10 codes for T2DM. All eligible patients were offered the EpxDiabetes service as part of their standard care under institutional policy and consented to receiving SMS messages/calls for health care communication. Patient recruitment and enrollment continues on a rolling basis, and at the time these data were collected, patient enrollment had taken place from August 2015 to February 2017. The population extended to adults >18 years old in the greater St. Louis Metropolitan area consisting of St. Louis City and St. Louis County. Both populations’ demographics are listed in Table 1.

Aggregate de-identified data on patient engagement, HbA1c, and FBG were provided by Epharmix, Inc, and their clinical partners for analyzing outcomes with permission by the community health care institution. To be eligible for HbA1c analysis, patients needed a pretrial or baseline HbA1c value obtained within 6 months prior to receiving the first EpxDiabetes message. Participants also needed to obtain a posttrial HbA1c value between 2 and 5 months after receiving their first EpxDiabetes message. Patients who did not respond to a single intervention message were excluded from analysis.

### Statistical Analysis

Patient HbA1c deltas were calculated as the difference between pre-intervention baseline HbA1c and most recent HbA1c since enrollment in EpxDiabetes. These values were averaged per individual patient to determine the overall population’s aggregate delta. Historical, pre-EpxDiabetes HbA1c deltas were calculated by subtracting the two most recent HbA1c values before receipt of EpxDiabetes messages. Statistical significance was defined as *P*<.05 by one-sample *t* test with a theoretical mean delta HbA1c of 0.0%, and a two sample *t* test comparison of historical change in HbA1c versus postimplementation change in HbA1c. Standard error of the mean (SEM) and 95% confidence intervals (CI) were calculated as well. Identical HbA1c analysis was also performed for a subgroup of patients with a pretrial baseline HbA1c >8%.

Patient-reported FBG data were obtained by querying the Epharmix server. Baseline FBG for each patient was calculated as the median of the first 3 patient-reported FBG values. To account for the variable weekly message frequency between individual patients, monthly FBG was determined for each patient by averaging four consecutive weekly FBG averages. Monthly FBG deltas were calculated by subtracting the patient’s baseline and monthly FBG values. Individual patient monthly FBG deltas were averaged together to calculate the average monthly FBG delta of the population. We compared the average fasting blood glucose pre- and post-EpxDiabetes using a two-tailed one-sample *t* test, with significance set at *P*=.05. We calculated the SEM for each monthly FBG deltas.

We defined weekly patient engagement rate as the proportion of total patients responding at least once per week to EpxDiabetes messages. Monthly patient engagement rate was determined by averaging four consecutive weekly engagement rates. Cumulative monthly patient engagement was calculated by averaging the monthly patient engagement. Gross response rate was defined by the number of messages responded to out of the total number of messages sent. Patients who did not respond to the initial consent message were excluded from engagement analysis.

We performed data analysis on Microsoft Excel 2016 and PRISM (GraphPad Software, 2016). Because of overtitration concerns, we found clinic providers aimed for optimizing FBG equivalent to an HbA1c cutoff of 8%. Therefore, analysis was performed for all patients and for the subset of patients with a baseline HbA1c >8%.

**Table 1:** St. Louis City and County residents’ demographic and income data from which the Epharmix population was recruited.

Characteristics			St. Louis City		St. Louis County
Population estimates, n			315,685		1,003,362
**Age in years, %**					
	18-65	61.2		55.2	
	>65	11		16.8	
**Gender, %**					
	Male	48.3		47.7	
	Female	51.7		52.3	
**Ethnicity, %**					
	Caucasian	43.9		69.5	
	African American	49.2		24.1	
Median household income 2011-2015, USD			$35,599		$59,755

## Results

### Patient Demographics

In total, 396 patients were consented and enrolled at a large St. Louis area health care institution who were already receiving standard of care treatment and education regarding their diabetes. In total, 79.3% (314/396) of patients in the community implementation consented to EpxDiabetes. The increased receptiveness of patients to use the service is perhaps due to EpxDiabetes being offered as part of their standard of care. Individual socioeconomic data were not able to be collected or analyzed for this particular implementation project. The majority of patients were adults from St. Louis City and County, and census data for socioeconomic status are reported as a corollary in Table 1 (US Census) [13].

### HbA1c Analysis

For analysis of the effect of EpxDiabetes on the population, immediate pre-implementation HbA1c values were obtained, on average, 2.0 months (60.0 days) before implementing EpxDiabetes. Just under half (45.4%, 166/366) had pre-implementation HbA1c values available. Patients obtained their posttrial HbA1c values, on average, 4.0 months after intervention start (63.9%, 106/166). Not all patients had HbA1c values measured during their routine standard of care pre- and post-EpxDiabetes usage, so analysis was limited to all HbA1c values available. Because EpxDiabetes was implemented in a community population, patients were at various stages of getting HbA1c measured, which often fell outside of the 4-month window. As such, during analysis we looked only at patients who had both a pre- and post-HbA1c value during the 4-month analysis period (33.3%, 106/318). This snapshot is as a result a random sampling of HbA1c across the entire population.

Prior to receiving the EpxDiabetes intervention, patients with HbA1c >5% demonstrated nonsignificant changes of -0.13% (SEM 0.11, 166/366) during the last 3 months, suggesting the standard of care was not sufficient to change underlying diabetes control. The immediate average pre-EpxDiabetes HbA1c for these patients was 8.89% (SEM 0.18). The average post-EpxDiabetes HbA1c was 8.17% (-0.72%, SEM 0.17, 95% CI -1.05 to -0.39; 106/166). The comparison of pre- and postintervention was statistically significant (*P*=.004).

**Figure 2:**
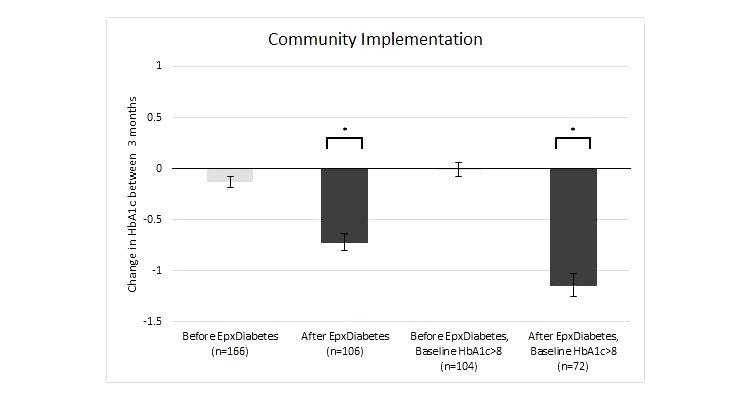
Change in HbA1c from pretrial baseline at 4 months from initiation (error bars represent SEM; *P<.05 for change from baseline).

A total of 22.6% (72/318) of patients had a baseline pre-implementation HbA1c >8%. For this group, prior to receiving the EpxDiabetes intervention, patients demonstrated nonsignificant changes of -0.005% (SEM 0.14, 104/318), respectively, during the prior 3 months. The immediate average pre-EpxDiabetes HbA1c was 9.81% (SEM 0.18). The average postimplementation HbA1c for this group was 8.66% (-1.15%, SEM 0.21, 95% CI -1.58 to -0.71, 72/106). The comparison of pre- and postintervention was statistically significant (*P*=.0018) (Figure 2).

### Fasting Blood Glucose Analysis

**Figure 3:**
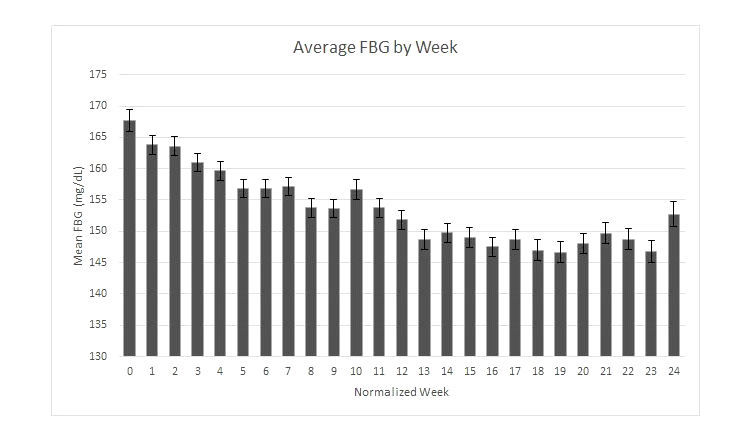
Average fasting blood glucose change over time (error bars represent SEM).

The average postimplementation FBG as reported on EpxDiabetes was significantly lower from month 2 on intervention through time of analysis (Table 2). The EpxDiabetes system was also able to identify and alert providers in real time to 395 total acute hypoglycemic (FBG<70) or hyperglycemic (FBG>400) events. These alert notifications resulted in 228 patient-initiated calls and 83 provider-initiated calls and interventions. For average FBG changes over time, 153 patients had been on the intervention for at least 6 months. Average monthly FBG values over the 6 months were significantly lower at each month when compared to the sample’s baseline average (Figure 3 and Table 2).

**Table 2:** Fasting blood glucose changes by month.

Month	1	2	3	4	5	6
Mean FBG (mg/dL)	160.4	154.3	149.8	146.2	146.6	148.4
SEM	3.037	2.678	2.455	2.398	2.468	3.43
Delta	N/A	-5.677	-10.19	-13.78	-13.41	-11.59
95% CI	N/A	-10.95 to -0.4017	-15.03 to -5.357	-18.51 to -9.047	-18.28 to -8.538	-18.37 to -4.813
*P* value (two tailed)	N/A	.035^a^	<.001^a^	<.001^a^	<.001^a^	.009^a^
N	308	247	223	189	169	153

^a^*P* ≤.05.

### Response Rate

The EpxDiabetes system conducted a total of 55,730 FBG assessments during the 6-month implementation for a total of 396 patients. There were 80 patients who did not respond to the initial consent message and therefore did not receive any further text messages during the 6-month period trial and were excluded from engagement analysis. Of the 316 consenting patients, 41 never responded to any subsequent messages (13.0%). Of those who responded to at least one message, EpxDiabetes had an average of 95.6% patient response rate to messages at least once per month and had an average 71.1% (range 64.8-79.3) response rate to messages at least once per week. A total of 83 patients revoked the use of EpxDiabetes service via the “opt-out” feature wherein a patient could text “STOP” to the system, thereby discontinuing any further messages, showing an absolute dropout of 21%. The average weekly dropout was 1.1% of the total. We had a gross response rate of 55.7% to all messages sent through 6 months.

## Discussion

### Principal Considerations

Glycemic control remains a difficult goal to achieve among patients with diabetes. Current strategies to achieve outpatient HbA1c control prove inadequate for a large proportion of patients [14,15]. Electronic health care tools involving phone communication or mobile phone apps have been utilized to improve glycemic control [8,9,16], but their cost and the unidirectional nature of communication reduces accessibility and/or effectiveness among low socioeconomic and education populations.

Our intervention, EpxDiabetes, is a novel phone call and SMS-based communication tool specifically designed to address these limitations. The intervention aims to accelerate HbA1c reduction by providing caregivers with actionable automatically triaged patient-reported FBG data to facilitate a bidirectional loop of patient-provider communication. The study population, primarily consisting of middle- to older-aged individuals with a low median income and educational attainment levels, reflects our goal to produce a universally accessible, affordable, and user-friendly health care tool [17]. The cost to provide EpxDiabetes is substantially lower than required for many specialized telemonitoring devices. Our implementation shows a statistically significant HbA1c reduction from baseline for patients with baseline HbA1c >8%. The drop in HbA1c is corroborated by a significant decrease in self-reported average fasting blood glucose. Among participants, this reduction in FBG results in a 10.9% increase in patients reporting FBG<130, implying that EpxDiabetes accelerates HbA1c control by maintaining FBG control longitudinally. Based on previous results from the United Kingdom Prospective Diabetes Study, our HbA1c reductions demonstrated with the community implementation program are similar to those achieved with pharmacologic treatments and would represent a 37% decrease in the risk of microvascular complications and a 21% reduction in the risk of any diabetes-related complication or death, suggesting that this bidirectional communication between patient and provider could have important clinical implications [18].

We attribute the results of EpxDiabetes to increased patient investment in self-health combined with active monitoring and titration by their care teams. The patient-reported FBG data allow providers to perform care-plan adjustments based on actionable data and receive rapid feedback on these adjustments. This closed reactive feedback loop allows EpxDiabetes to complement treatment plans and accelerate glycemic control by optimizing current medications.

In contrast to FBG diaries and several electronic health tools utilizing specialized equipment and mobile phone apps [8,10], EpxDiabetes demonstrates high patient engagement. With over 85% of participants continuing to communicate with the system at least 1 month until their respective conclusions at 6 months, EpxDiabetes circumvents engagement limitations seen with other electronic interventions to keep patients engaged long term.

We attribute the high weekly and longitudinal patient engagement to two components of EpxDiabetes: the regularly scheduled proactive messages may serve as a “buddy,” helping patients establish a habit of checking their glucose the same time every day. The bidirectional design also encourages involved providers to call following dysglycemic events, helping patients feel more connected to their health care providers, and further incentivizing patient engagement with the system [19-21]. These factors may explain the higher engagement rate for the intervention group compared to the nonintervention group. The overall findings suggest that EpxDiabetes provides a more engaging alternative to FBG diaries for both short-term and long-term FBG monitoring.

### Study Limitations and Next Steps

Overall, the community implementation demonstrated encouraging trends in HbA1c and FBG reduction, particularly in the HbA1c >8% population. Given the positive outcomes associated with actively engaged patients and care teams, further educational messages to encourage lifestyle behavior modifications are an avenue worth exploring in future iterations of the system. This first report demonstrates the capability of EpxDiabetes to maintain high engagement with patients and impact a population change in HbA1c over a short time period with a simple low overhead system. We will report the results after 1 year to see if the system is able to maintain this HbA1c drop. Further study at 1 year and beyond will provide better data following increased enrollment and more time for patients to get their regularly scheduled HbA1c tested. Despite this limitation, the random sampling does suggest a change in HbA1c, when considering the same patients did not show significant change prior to use of EpxDiabetes, lending pre-post evidence to the hypothesis that EpxDiabetes was instrumental, at least in part, for the improvement in glycemic control. Furthermore, we are currently conducting a phase II/III, randomized controlled trial based on the results with a larger study size to characterize EpxDiabetes’ effect on patient outcomes as compared to a simultaneous standard of care group.

### Conclusion

The results of this study suggest that EpxDiabetes is an inexpensive, low-risk, noninvasive intervention that can be implemented in a variety of settings to accelerate glycemic control for patients with T2DM with baseline HbA1c >8%. The results merit future investigation of the long-term effects of EpxDiabetes on patient health outcomes.

## Abbreviations

CI: confidence interval
FBG: fasting blood glucose
HbA1c: glycated hemoglobin
SEM: standard error of the mean
SMS: short message service
T2DM: type 2 diabetes mellitus
